# 869 But It’s a Dry Heat: Exploring the Relationship Between the Environment and Burn Resuscitation

**DOI:** 10.1093/jbcr/iraf019.400

**Published:** 2025-04-01

**Authors:** Stacey Richerbach, Claudia Islas, Karen Richey, Kevin Foster

**Affiliations:** Diane & Bruce Halle Arizona Burn Center; Diane & Bruce Halle Arizona Burn Center; Diane & Bruce Halle Arizona Burn Center; Diane & Bruce Halle Arizona Burn Center

## Abstract

**Introduction:**

Over five years, our Burn Center recorded high resuscitation rates, averaging 6.5mL/kg/TBSA. Despite high rates, complications such as fluid overload, organ dysfunction, and other adverse events were low and did not suggest a greater risk of harm to others. As we sought to identify a cause for high fluid resuscitation rates, conventional wisdom lent the notion that our arid environment may result in patients presenting initially with a low hydration status, ergo increased fluid demand. The purpose of this study was to explore the relationship between our locale and burn resuscitation, and to identify factors associated with resuscitation volume.

**Methods:**

This was a retrospective chart review of patients admitted over a five-year period requiring resuscitation. Patients with incomplete records and those who expired within 48 hours of injury were excluded as these prevented adequate outcomes assessment and fluid management. Variables analyzed included admission serum creatinine (sCr) and hematocrit (HCT), in-to-out ratio (IOR), total volume given, bolus volume given, hourly rate of fluid administered, outdoor temperature, and the use of CRRT. When examining HCT, individuals were stratified by gender into high (HCT-H), within normal limits (WNL), or low (HCT-L) groups. Descriptive statistics and Pearson correlation were completed.

**Results:**

A total of 138 adult patients with a burn TBSA >20% were evaluable. Of these, 87 (63%) received pre-hospital fluids (PHF). There was a moderate negative correlation between PHF and HCT-L (p <.05). There was no correlation between PHF and HCT-WNL or HCT-H did not correlate with PHF. Overall, 30% of patients presented with a high HCT. Of HCT-H, there was a positive correlation with both bolus volume (p <.01) and total volume given (p=.003) during resuscitation. HCT category did not correlate with IOR or outdoor temperature. Patients with a higher sCr had a higher IOR (p <.0001) and CRRT was more likely in the first 48h (p < 0.000001). Patients with higher IOR were more likely to have CRRT in the first 48h (p 0.006), however, hourly fluid rate was not significantly different between those who did or did not undergo CRRT.

**Conclusions:**

While hydration status cannot not be determined on sCr and HCT alone, particularly in the face of initial post-injury fluid shifts, our findings suggest that sCr and HCT may be useful when evaluating the appropriateness of resuscitation volumes. Further research is warranted as this was a single-center study with limited sample size. Also, results may be confounded by rates at which sCr and HCT equilibrate following fluid administration. A larger multi-center trial, across a variety of climates is needed to better examine this phenomenon.

**Applicability of Research to Practice:**

When evaluating resuscitation practice, consideration should be given to locale and patient-specific findings.

**Funding for the Study:**

N/A

